# Giant cell tumor of the maxilla: an unusual neoplasm

**DOI:** 10.11604/pamj.2020.36.342.21919

**Published:** 2020-08-25

**Authors:** Soukayna Bahbah, Karima El Harti, Wafaa El Wady

**Affiliations:** 1 Department of Oral Surgery, Faculty of Dentistry, Mohammed V University, Rabat, Morocco

**Keywords:** Giant cell tumor, multinucleated giant cell, maxilla, curettage

## Abstract

Giant cell tumors (GCT) of the bone are uncommon primary bone neoplasms that occur mainly in the epiphyses of long bones. Their incidence in craniofacial skeleton is rare, particularly in the maxilla. We report a case of a 12-year-old patient with a GCT of the left maxilla, who underwent a surgical excision of whole mass, and showed no recurrence one year after intervention.

## Introduction

The giant cell tumor (osteoclastoma) is a benign, locally invasive lesion of the bone [1]. Giant cell tumor (GCT) typically affects the meta-epiphyseal region of long bones, mainly the distal femur and the proximal tibia [2] with a high incidence in female in the 2^nd^ and 3^th^ decades of life. This lesion is uncommon in the craniofacial skeleton; the majority of them occur in the sphenoid, ethmoid and temporal bones. A review of the literature showed that GCT of the maxilla has been seldom encountered [3]. To date, only a few reports of GCT arising from the craniofacial skeleton have been published. In this case report we share our experience of managing one such extremely rare case of giant cell osteoclastoma of maxilla. The learning objective of this article is to present the diagnosis and management of this rare lesion along with short discussion on the characteristics inherent to GCT.

## Patient and observation

**Figure 1:**
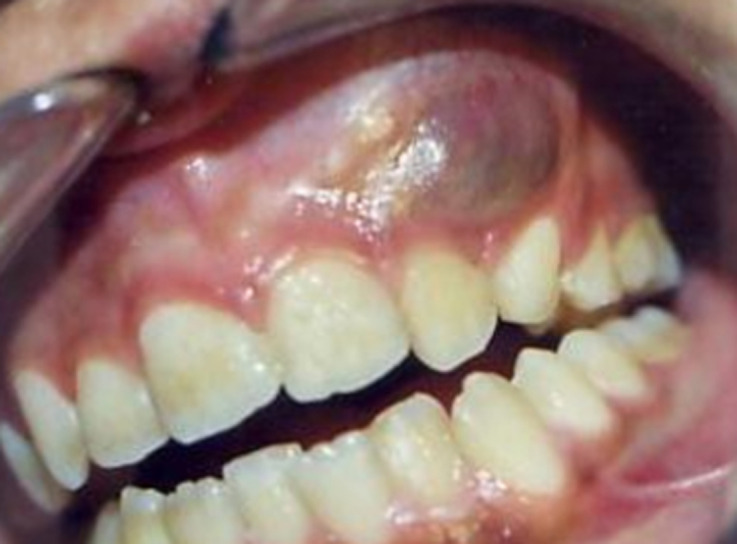
red-purple prominent lesion involving left maxilla that appears swollen and edematous, causing deformity of the jaw

A 12-year-old girl was referred to Department of Oral Surgery, with a complaint of pain and swelling in the left of maxilla that appeared 3 months ago. The swelling was slowly progressive, associated with pain. However, the patient did not refer any motor or sensory deficit. There was no family history of similar swelling. Physical examination revealed a left maxillary swelling. There was no facial palsy. No cervical lymph node enlargement was seen. The oral examination showed a 4 cm x 5 cm, tender, compressible mass in the left maxilla, from the lateral incisor to the second premolar teeth. The tumor surface was smooth and red-purple (Figure 1). Several teeth were involved and displaced in the tumor mass. We noted the absence of canine. Vitality tests proved negative on the central and lateral incisor. Hight mobility of lateral incisor was noted.

**Figure 2:**
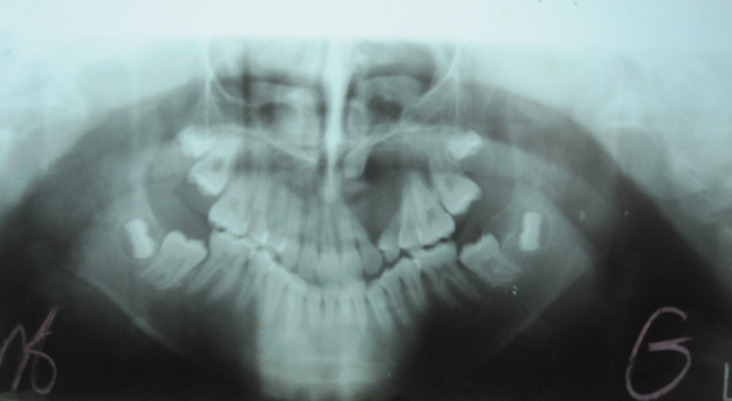
preoperative orthopantomography radiography, showing an expansive lesion of the anterior left maxilla involving the nasal cavity and extending superiorly closely to the floor of the maxillary sinus. The canine is impacted

**Figure 3:**
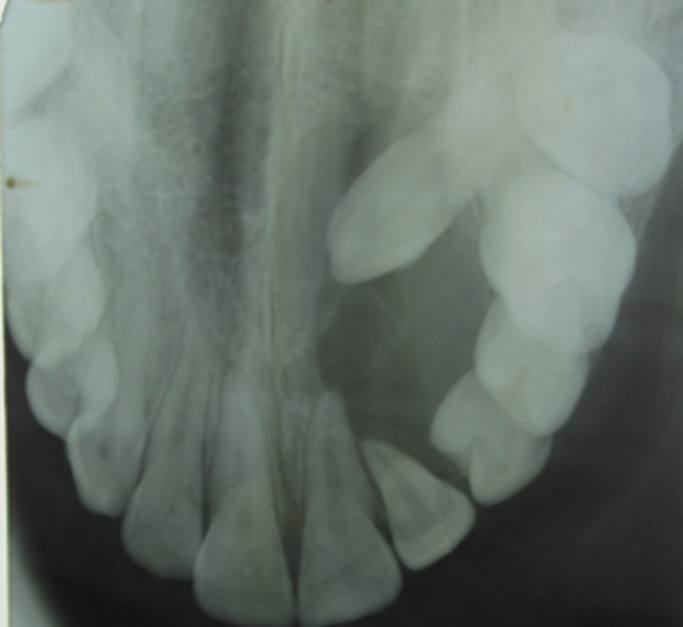
preoperative occlusal radiography

**Figure 4:**
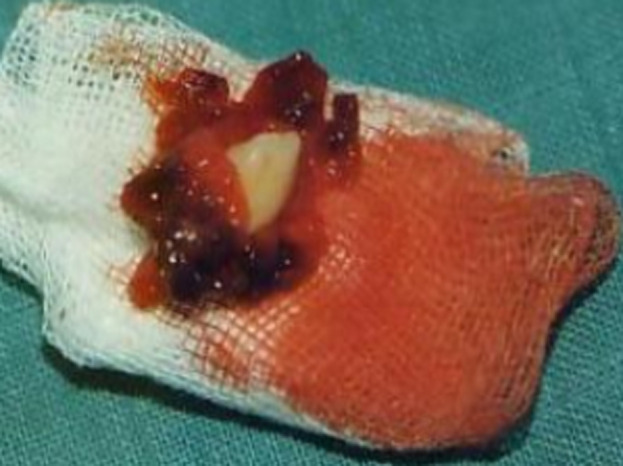
clinical aspect of the lesion

**Figure 5:**
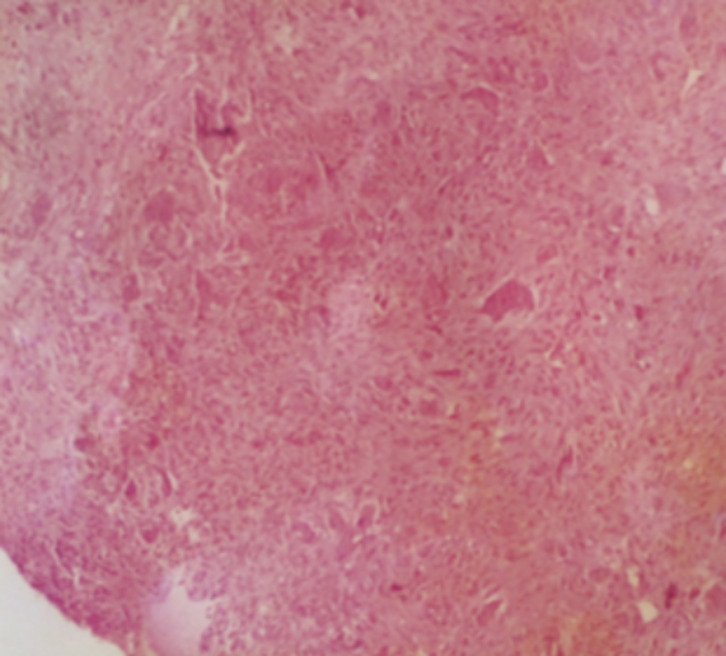
photomicrograph (hematoxylin-eosin stain) showing multiple multinucleate giant cells (arrows) in a spindle cell stroma

**Figure 6:**
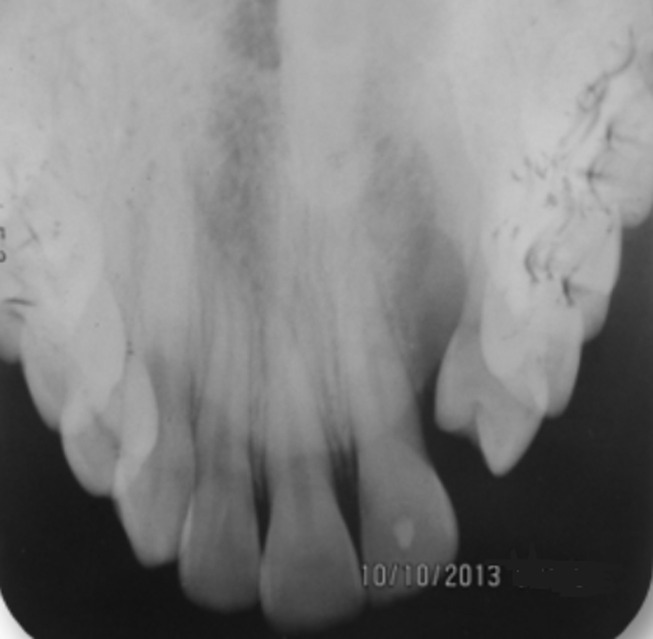
1 year postoperative follow up showing bone formation

Orthopantomography and occlusal radiography showed a wide osteolytic area of the anterior left maxilla, extending from the first permanent molar to the central incisor (Figure 2, Figure 3). The canine was impacted. No root resorption was observed. The radiolucency was closely related to maxillary sinus and involving the nasal cavity. Haematological investigations showed normal serum calcium, phosphorus and parathormone (PTH) levels. The provisional diagnosis of benign tumor of the maxilla was made. The patient underwent excision and curettage of the mass with extraction of the canine and lateral incisor tooth (Figure 4). The wound was closed with interrupted sutures. The post-operative histopathological report revealed multinuclear giant cells scattered randomly throughout the cellular and fibrous vascular-rich tissue. New bone formation and granulation tissue rich in mononuclear inflammatory cells was revealed. The giant cells were multinucleated with bland-appearing nuclei, and the background stromal cells displayed no evidence of atypical mitoses (Figure 5). A diagnosis of giant cell tumor was established. During a 1-year serial clinical and radiological follow-up, there was no evidence of recurrence (Figure 6). The facial contour and masticatory function were well-preserved.

## Discussion

GCT is a true neoplastic process originating from the undifferentiated mesenchymal cells of the bone marrow [4]. They are characterized by a profuse multinucleate giant cell scattered throughout the stroma of mononuclear cells. These giant cells have some similarity with osteoclasts, and so are called osteoclastoma [1,5]. GCT is generally considered as benign [6] but severe bony destruction may result occasionally depending on the location and clinical presentation of the tumor, making tumor management very challenging [2]. GCTs are usually mono-ostotic, however they may occasionally present in a polyostotic form, which is usually of a high grade. The incidence of the GCT is 5% of all primary bone neoplasms [7]. Although only 2 to 4% of all GCTs occur in the head and neck [8]. The case report described here has its presentation in the left side of maxilla involving partially nasal cavity. There are few reports found in the literature describing involvement of jaw bone by GCT. This adds one more aspect in diversity of clinical and biological behaviour of GCT in maxillofacial region. Clinically, the lesion usually occurs in young adults below 20 years of age. There seems to be slight preponderance towards females [9]. The above description of the clinical age and sex predilection is seen in our case report. The GCT presents as slow growing tumor, with diverse symptoms depending on the location of primary lesion; symptoms include swelling, pain, epistaxis, neurological deficits, proptosis, visual defects, tinnitus, and malocclusion [10]. In this case, GCT arising from the maxilla caused pain and tooth mobility.

The pathogenesis of GCT has added to diagnostic dilemma. Lichtenstein in 1950 postulated that GCT is a manifestation of altered haemodynamics. The pressure secondary to circulatory disturbance leading to congestion leading to bone resorption, with deposition of fibrous connective tissue, osteoid and new bone. A second hypothesis was given by Biesecker et al. [11] where they proposed that a primary lesion initiates an osseous, arteriovenous malformation and thereby creates, via its haemodynamic forces, a secondary reactive lesion of bone. The most commonly accepted theory of Hillerup and Hjorting-Hansen [12] suggested that GCT, Central Giant Cell Granuloma (CGCG), Traumatic Bone Cyst (TBC) were all related lesions. Minute trauma or the presence of unidentified small aneurysmal enlargements may result in intramedullary bleeding leading to haematoma. If the blood supply is lost TBC may develop. If only small vessels or low pressure is present then capillary and endothelial proliferation occurs resulting in CGCG. If circulation is maintained, creating high pressure, large pools of blood are formed and GCT results. The clinical case did not give any history of trauma and hence this cannot be assigned as the cause of pathogenesis. Radiological findings vary from small unilocular lesion to large multilocular lesion with well or ill-defined borders. It may also be associated with cortical bone perforation and root resorption [13]. A CT scan can provide a detailed assessment of GCT, showing the soft tissue mass of the lesion, cortical perforation, amount of bony destruction, and extension toward important adjacent anatomic structures [2]. MRI is superior to CT in delineating the extent of soft tissue tumor because of its improved contrast resolution [2]. Bone scintigraphy shows increased radionuclide uptake in the majority of GCTs [2]. In the present case panoramic radiograph showed unilocular osteolytic lesion with tooth displacement and root resorption.

GCT shows many clinical and radiological features in common with other bony lesion of the jaw, including giant cell granuloma, aneurismal bone cyst, fibro-osseous lesions, cherubism, brown tumor of hyperparathyroidism and malignant neoplasm of the jawbone such as sarcoma [14]. These lesions can be difficult to distinguish from one another, emphasizing the need for careful and thorough clinical, pathological and radiological correlation [15]. This is particularly true of brown tumor of hyperparathyroidism, which can be indistinguishable from GCT at pathological analysis. Laboratory analysis should be performed to exclude this possibility in all cases. In our case serum calcium, phosphorus and parathormone (PTH) were within normal limits, ruling out the diagnosis of brown tumor [16]. The real problem diagnosis seems to be between GCT and CGCG. The frequently described histological differences between the GCT and CGCG are; the rounded, larger giant cells which are uniformly dispersed with an increased number of nuclei which tends to aggregate centrally in the GCT, fresh hemorrhage and hemosiderin deposits and inflammatory component being found more commonly in the CGCG than in the GCT, osteoid or new bone formation and increased number of spindled fibroblasts with areas of fibrosis are more in the CGCG and the presence of necrotic areas in the GCT, but not in the CGCG [17].

The final diagnosis is established only on the basis of a biopsy. Histologically, GCTs are composed of multinucleated giant cells in a vascular stroma of epitheloid or spindle-shaped mononuclear cells, with peripheral osteoid formation [10,18]. The similar histopathological report in our case report supports its final diagnosis as GCT. Various treatment modalities have been used in management of GCT. The treatment of choice is surgical excision [4]. Regardless of the site of presentation, partial resection or curretage results in a recurrence rate of up to 70%, whereas recurrence after wide resection is about 7%. However, resection must be done only in case of multiple recurrences or extension to overlying tissues [19]. Radiation has been used as a therapeutic modality, but the subsequent development of sarcoma is possible and has been reported [20]. Other treatment modalities including cryotherapy, chemotherapy, intralesional steroids, calcitonin, interferon alfa and curretage with adjuvant agents (phenol and methyl methacrylate) have been tried, but have yielded less effective results [1,4]. In our case, the lesion was managed by excision and curettage.

## Conclusion

In conclusion, GCT arising from the maxilla is a rare disease whose diagnosis is difficult. Imaging and clinical features are often not sufficient to make the diagnosis. Therefore, the possibility of GCT should be included in the differential diagnosis of a bony lesion of craniofacial bones until a final diagnosis is made using a permanent pathologic specimen. Wide complete excision is required since incomplete excision results in a high incidence of recurrence.

## References

[1] Algawahmed H, Turcotte R, Farrokhyar F, Ghert M (2010). High-speed burring with and without the use of surgical adjuvants in the intralesional management of giant cell tumor of bone: a systematic review and meta-analysis. Sarcoma.

[2] Gino Marioni, Rosario Marchese-Ragona, Luca Guarda-Nardini, Roberto Stramare, Elia Tognazza, Filippo Marino (2006). Giant cell tumour (central giant cell lesion) of the maxilla. Acta Otolaryngol.

[3] Frederick FJ, Stewart IF, Worth AJ (1980). Giant cell lesion of the maxilla. J Otolaryngol.

[4] Ashwani Sethi, Passey JC, Sumit Mrig, Deepika Sareen, Prashant Sharma (2006). Giant cell tumour (osteoclastoma) of the zygoma: an extremely unusual neoplasm. Acta Otolaryngol.

[5] Schajowicz F (1961). Giant-cell tumors of bone (osteoclastoma): a pathological and histochemical study. J Bone Joint Surg Am.

[6] Leonard J, Gökden M, Kyriakos M,Derdeyn CP, Rich KM (2001). Malignant giant-cell tumor of the parietal bone: case report and review of the literature. Neurosurgery.

[7] Ferraz DFCD, Santos CATD, Costa VHF, Souza AMG, Lima PRG (2016). Giant-cell tumor: analysis on the importance of early diagnosis and the epidemiological profile. Rev Bras Ortop.

[8] Anjana Agrawa, Anusha Shukla (2016). Osteoclastoma of maxilla a rare case. Indian J Otolaryngol Head Neck Surg.

[9] Auclair LP, Arendt DM, Hellstein JW (1997). Giant cell lesions of the jaws: surgical pathology (fibroosseous diseases). Oral Maxillofac Surg Clin North Am.

[10] Se Ra Park, Sa Myung Chung, Jae-Yol Lim, Eun Chang Choi (2012). Giant cell tumor of the mandible. Clin Exp Otorhinolaryngol.

[11] Biesecker JL, Marcove RC, Huvos AG, Miké V (1970). Aneurysmal bone cysts. Cancer.

[12] Hillerup S, Hjorting-Hansen E (1978). Aneurysmal bone cyst simple bone cyst, two aspects of the same pathological entity?. Int J Oral Surg.

[13] Arun Dehadaray, Maitri Kaushik, Prasun Mishra, Vikrant Sagar (2013). Giant cell tumour of mandible: report of a rare case. Indian J Otolaryngol Head Neck Surg.

[14] Salzer-Kuntschik M (1998). Differential diagnosis of giant cell tumor of bone. Verh Dtsch Ges Pathol.

[15] Lang S (2008). Differential diagnosis of giant cell-rich lesions of bone. Der Pathologe.

[16] Kazunari Karakida, Yoshihide Ota, Takayuki Aoki, Tadashi Akamatsu, Hiroshi Kajiwara, Kenichi Hirabayashi (2010). Multiple giant cell tumors in maxilla and skull complicating Paget´s disease of bone. Tokai J Exp Clin Med.

[17] Giri G V V, Sukumaran G, Ravindran C, Narasimman M (2015). Giant cell tumor of the mandible. J Oral MaxillofacPathol.

[18] Wuelling M, Engels C, Jesse N, Werner M, Kaiser E, Delling G (2002). Histogenesis of giant cell tumors. Pathologe.

[19] Deepak Kulkarni, Lakshmi Shetty, Meena Kulkarni, Bela Mahajan (2013). Extensive giant cell tumour of the mandible: a case report with review. J Maxillofac Oral Surg.

[20] Jimmy Caudell J, Matthew Ballo T, Gunar Zagars K, Valerae Lewis O, Kristin Weber L, Patrick Lin P (2003). Radiotherapy in the management of giant cell tumor of bone. Int J Radiat Oncol Biol Phys.

